# Supplementary material for: The Structure of Stable Cellulolytic Consortia Isolated from Natural Lignocellulosic Substrates
Source: Int J Mol Sci. 2022 Sep 15;23(18):10779. doi: 10.3390/ijms231810779 (PMC9501375; doi:10.3390/ijms231810779)

Figure S1. Phenotype of the four cellulolytic consortia. From left to right: OS2 (oat straw), OS4 (oat straw), SD4 (pine sawdust), BL6 (birch leaf litter). Each consortia is presented by two tubes.

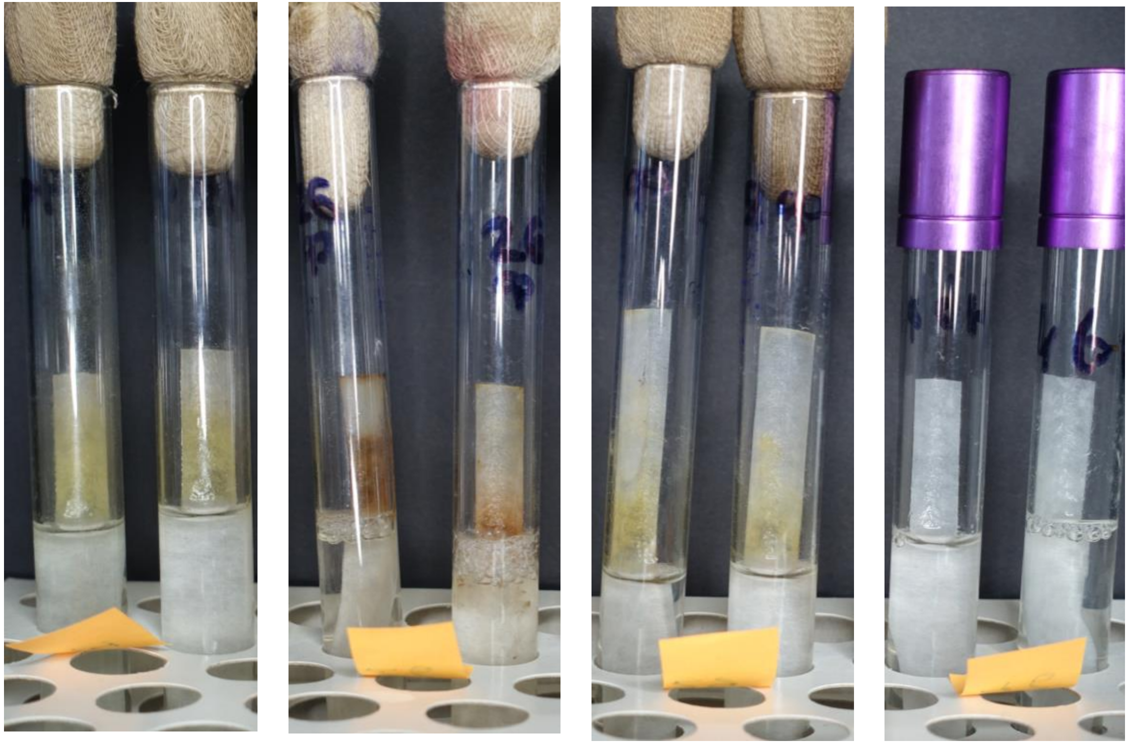

OS2 OS4 SD4 BL6

Figure S2. Heatmap of the most abundant eukaryote phylotypes on the genus level in the four consortia based on ITS sequencing data

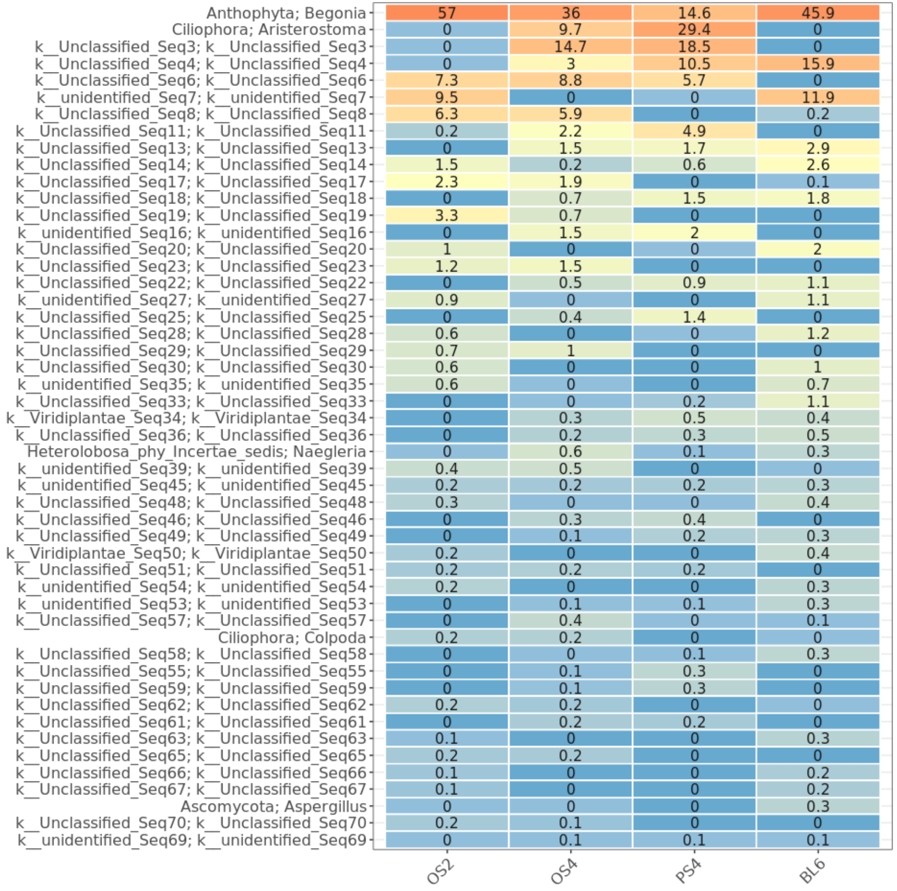

Figure S3. Beta-diversity of the four consortia (OS2, OS4, PS4, BL6) accessed by 3 visualization approaches – Bray-Cutris, UniFrac and Weighted UniFrac, based on 16S rRNA gene libraries

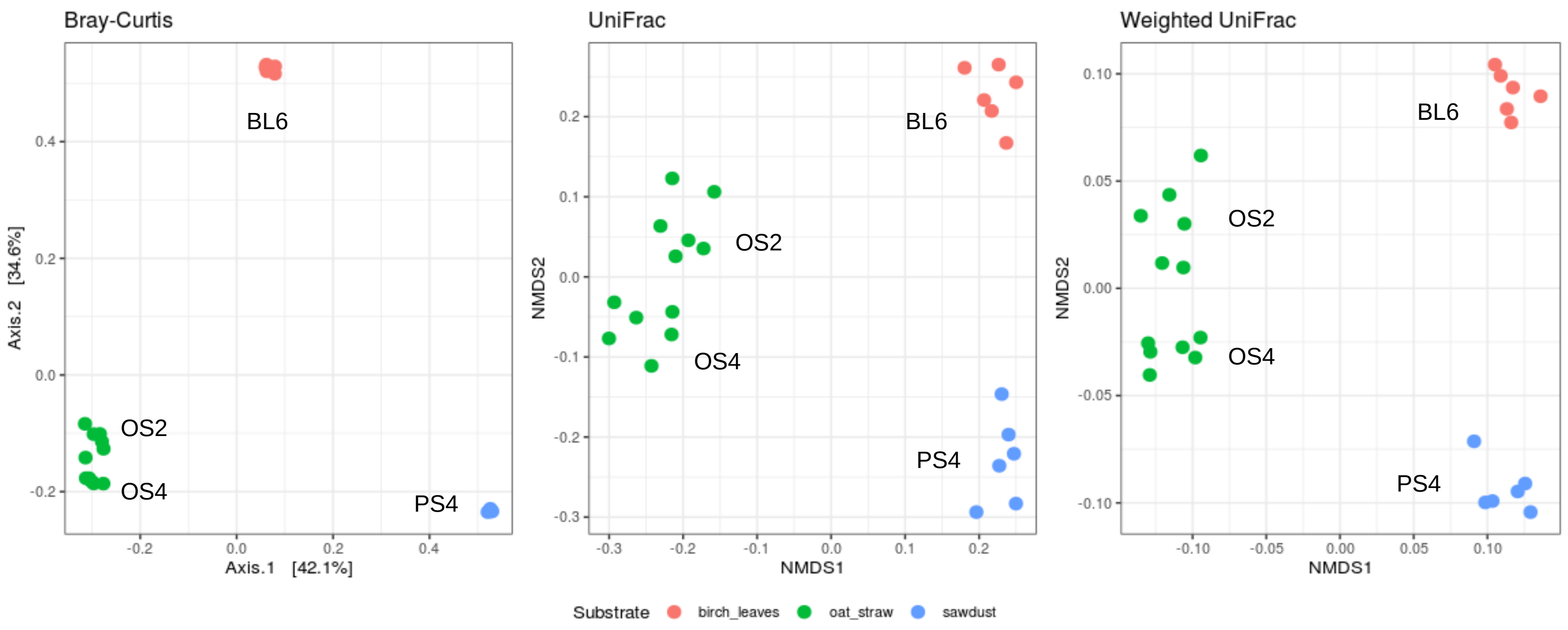

Figure S4. Heatmap of the most abundant bacterial phylotypes on the phylum level in the four cellulolytic consortia (OS2, OS4, PS4, BL6). Relative abundance given in % of the read count of each consortium, red for maximal, and green for minimal values

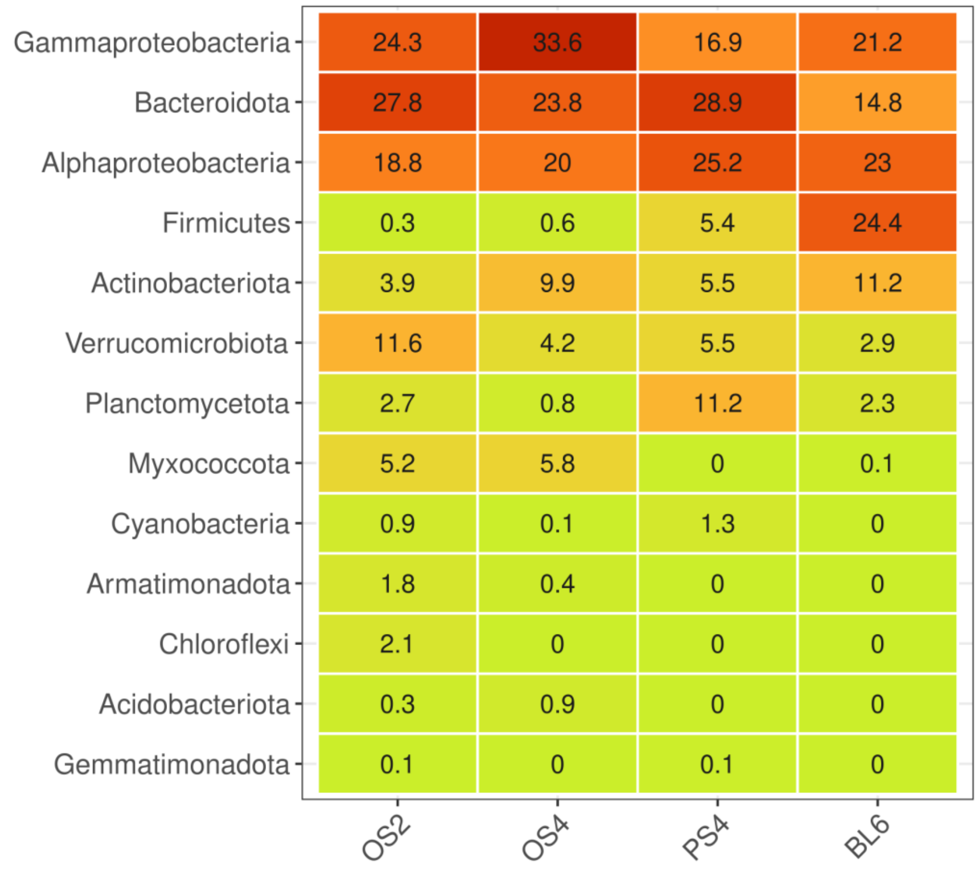

Figure S5. Dispersion of phylotype count between consortia

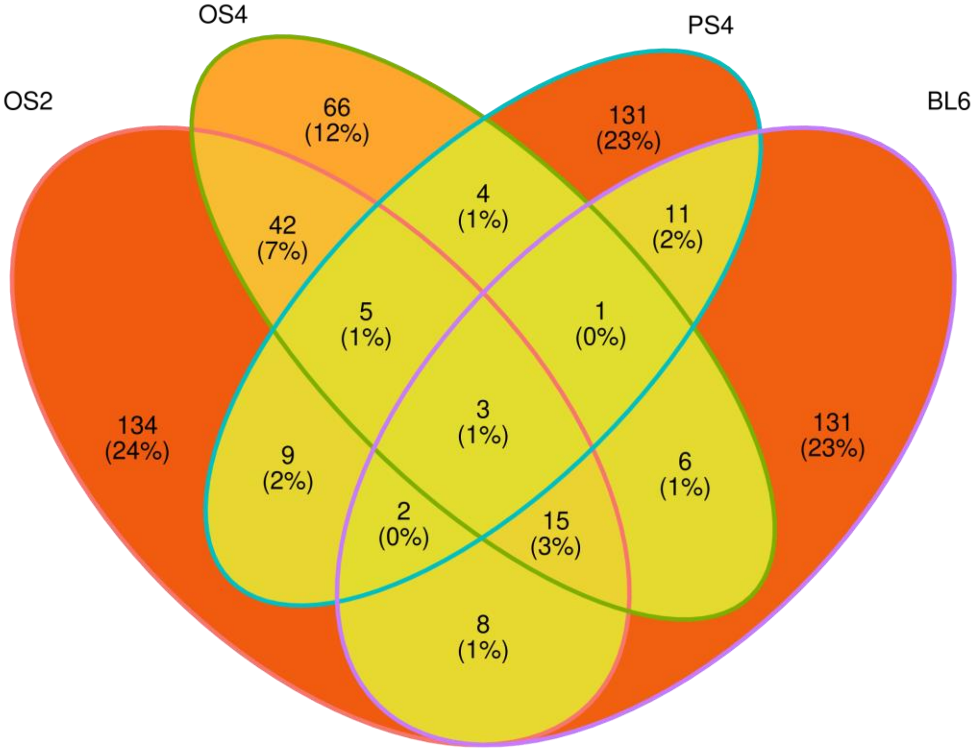

Figure S6. Analysis of balances: PhILR transformation between consortia OS2-OS4. (a) common phylotypes, (b) balance values, (c) balances on the dendrogram, (d) heatmap of the 30 most abundant bacterial phylotypes

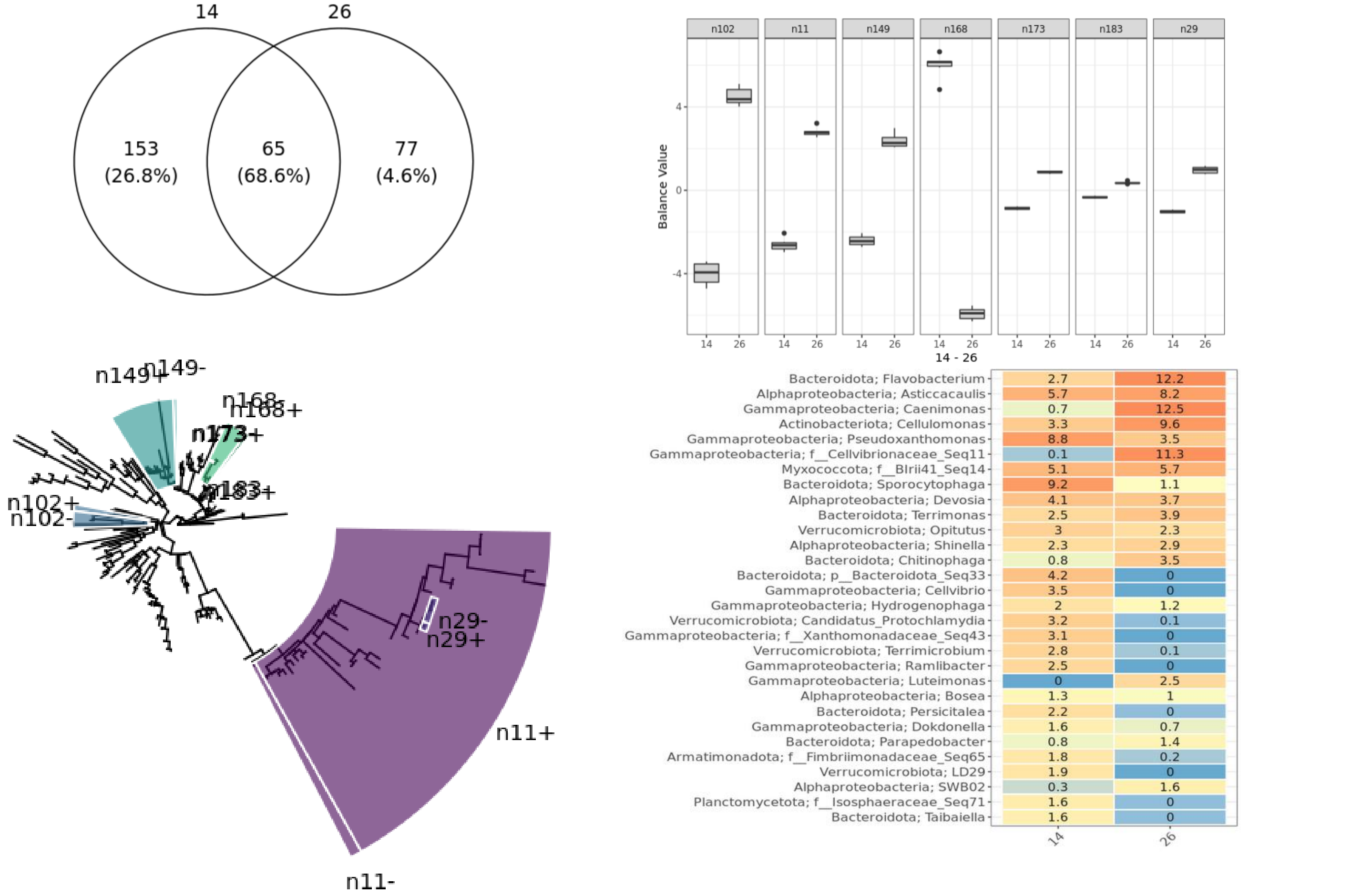

Figure S7. Analysis of balances: PhILR transformation between consortia OS4-PS4. (a) common phylotypes, (b) balance values, (c) balances on the dendrogram, (d) heatmap of the 30 most abundant bacterial phylotypes

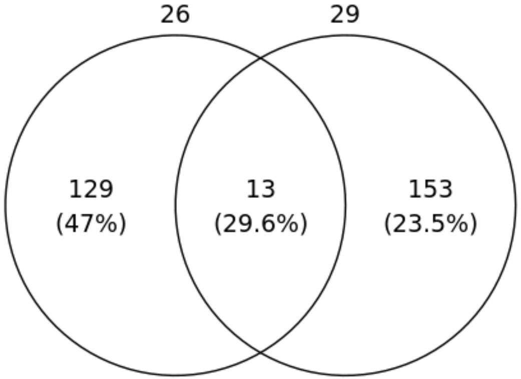

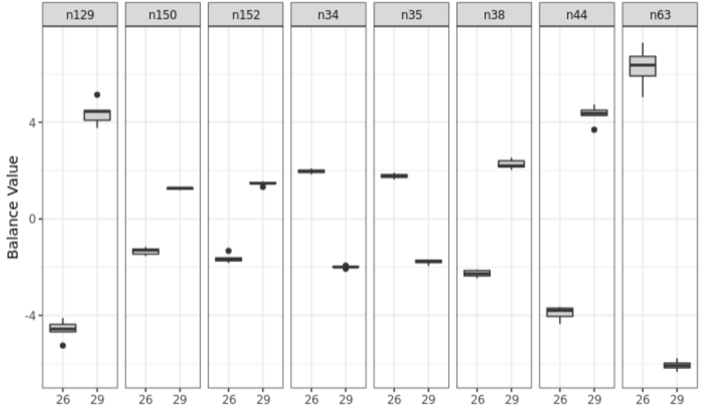

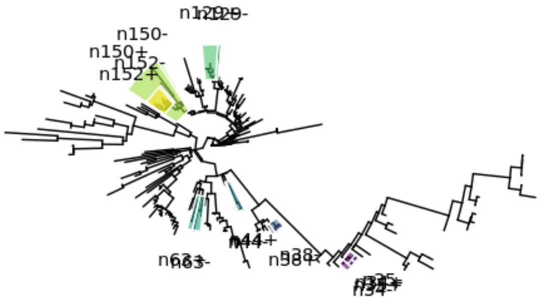

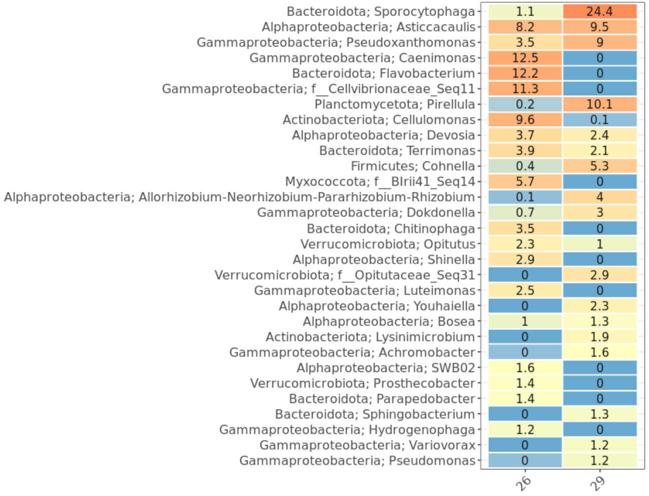

Figure S8. Analysis of balances: PhILR transformation between consortia OS4-BL6. (a) common phylotypes, (b) balance values, (c) balances on the dendrogram, (d) heatmap of the 30 most abundant bacterial phylotypes transformation between consortia.

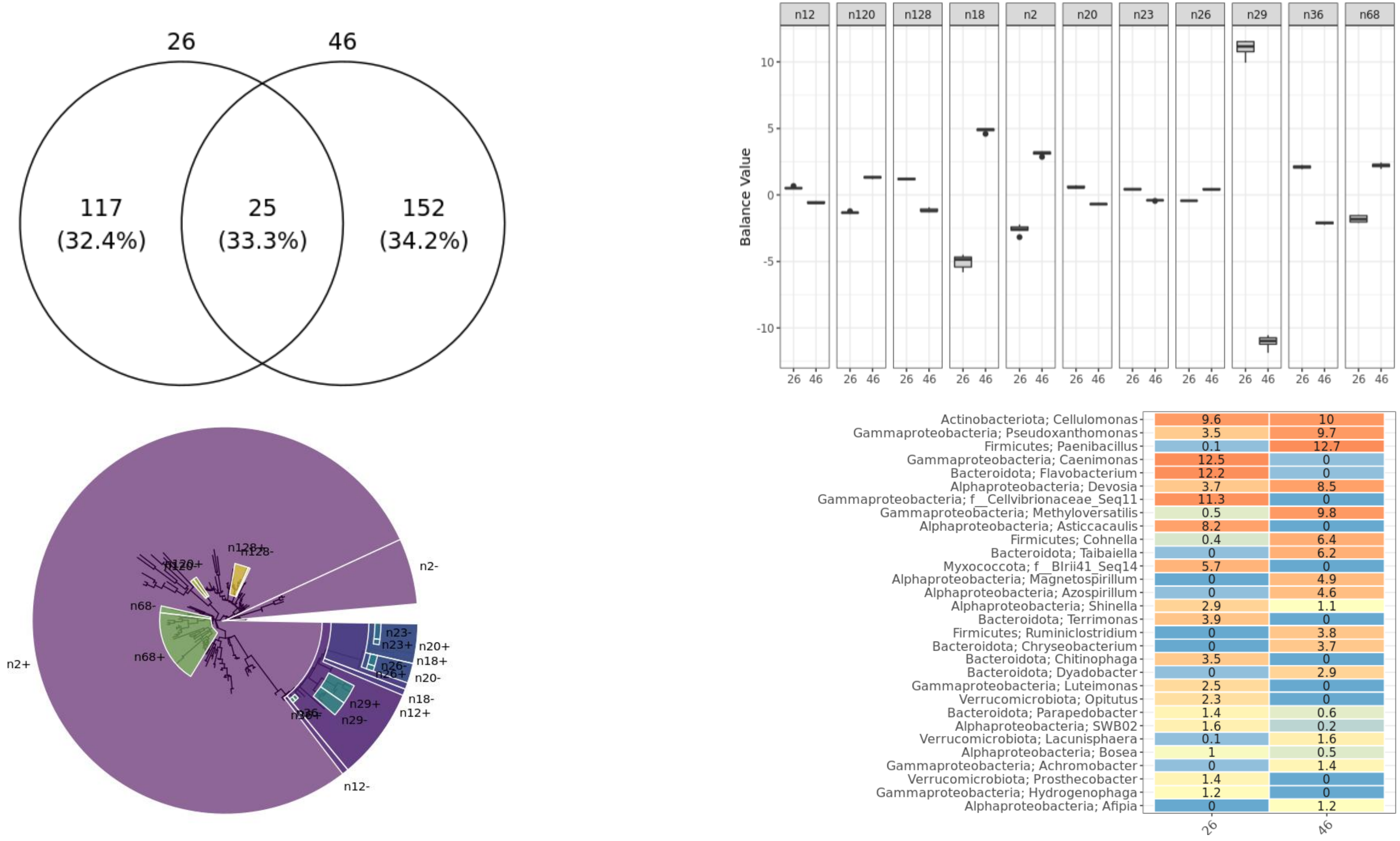

Figure S9. Analysis of balances: PhILR transformation between consortia PS4-BL6. (a) common phylotypes, (b) balance values, (c) balances on the dendrogram, (d) heatmap of the 30 most abundant bacterial phylotypes

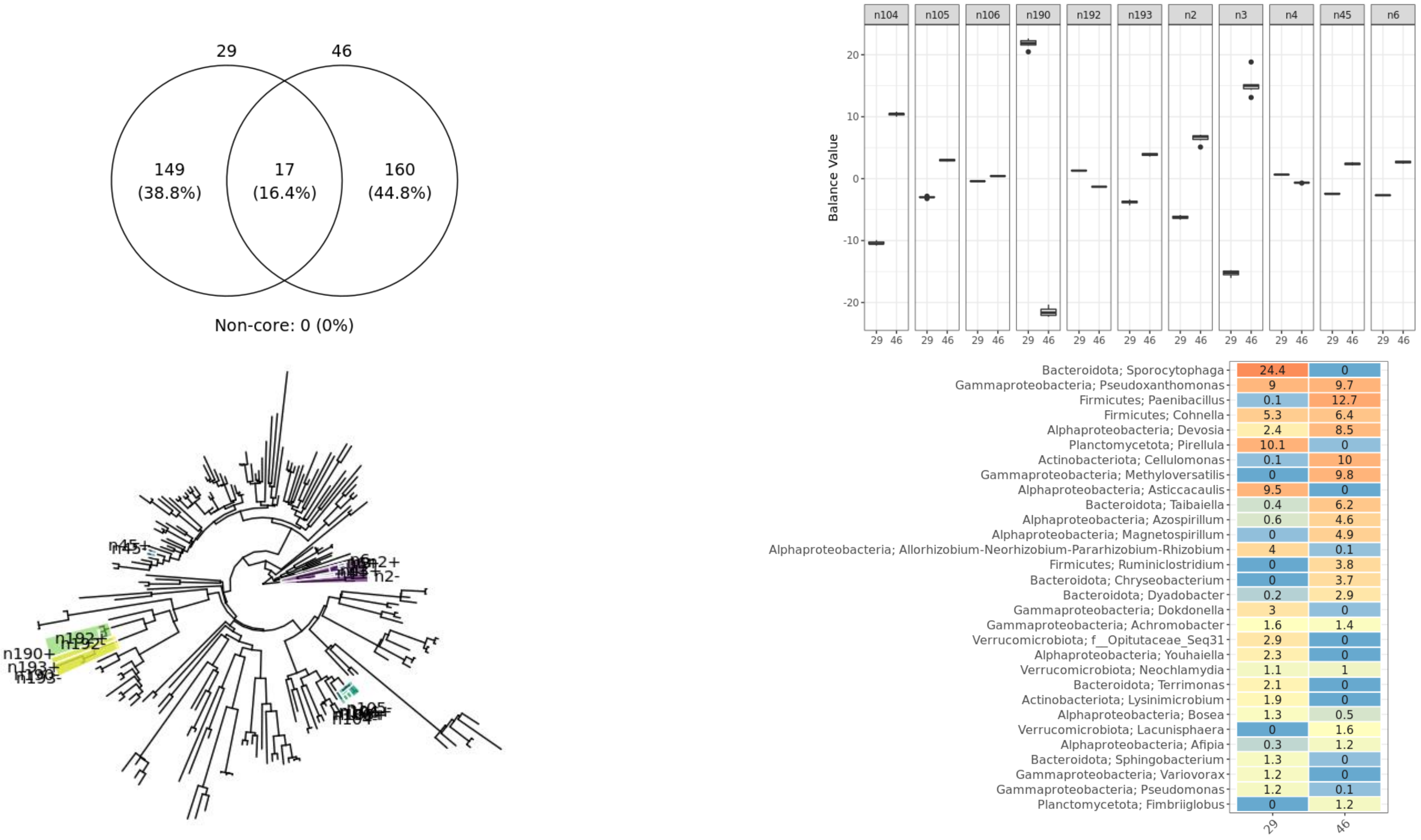

Figure S10. The most often cooccurring CAZy modules in the genes from cellulolytic consortia

CAZy	
CBM48,GH13	43
GH5,GH9	38
GH43,GH51	13
GT2,GT4	12
GH13,GH31	6
GH18,GH5,GH9	3
GH5,GH66,GH9	2
GH94,GT84	2
CBM20,GH13	2
CBM5,GH18	2
CBM6,GH43	2
GH6,GH82	2
AA10,CBM73	2
GH4,GT4	2
GH94,GT36,GT84	1
GH94,GT36	1
AA10,CBM15,CBM73,GH18	1
GH48,GH5,GH9	1
GH33,GH5,GH9	1
GH20,GH33	1
GH13,GH77	1
CBM42,GH54	1
CBM15,GH18,PL8	1
GH31,GH97	1

Figure S11. Diversity of GH and CBM families across all consortia. OS2: purple; OS4: blue; PS4: green; BL6: yellow.

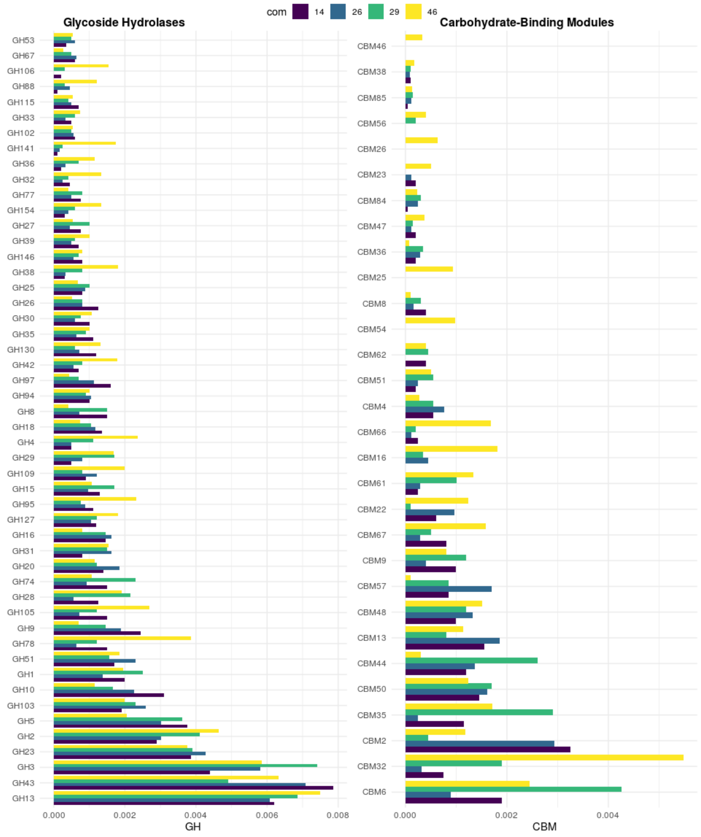

Figure S12. Example of the taxonomic unevenness of KEGGS between consortia

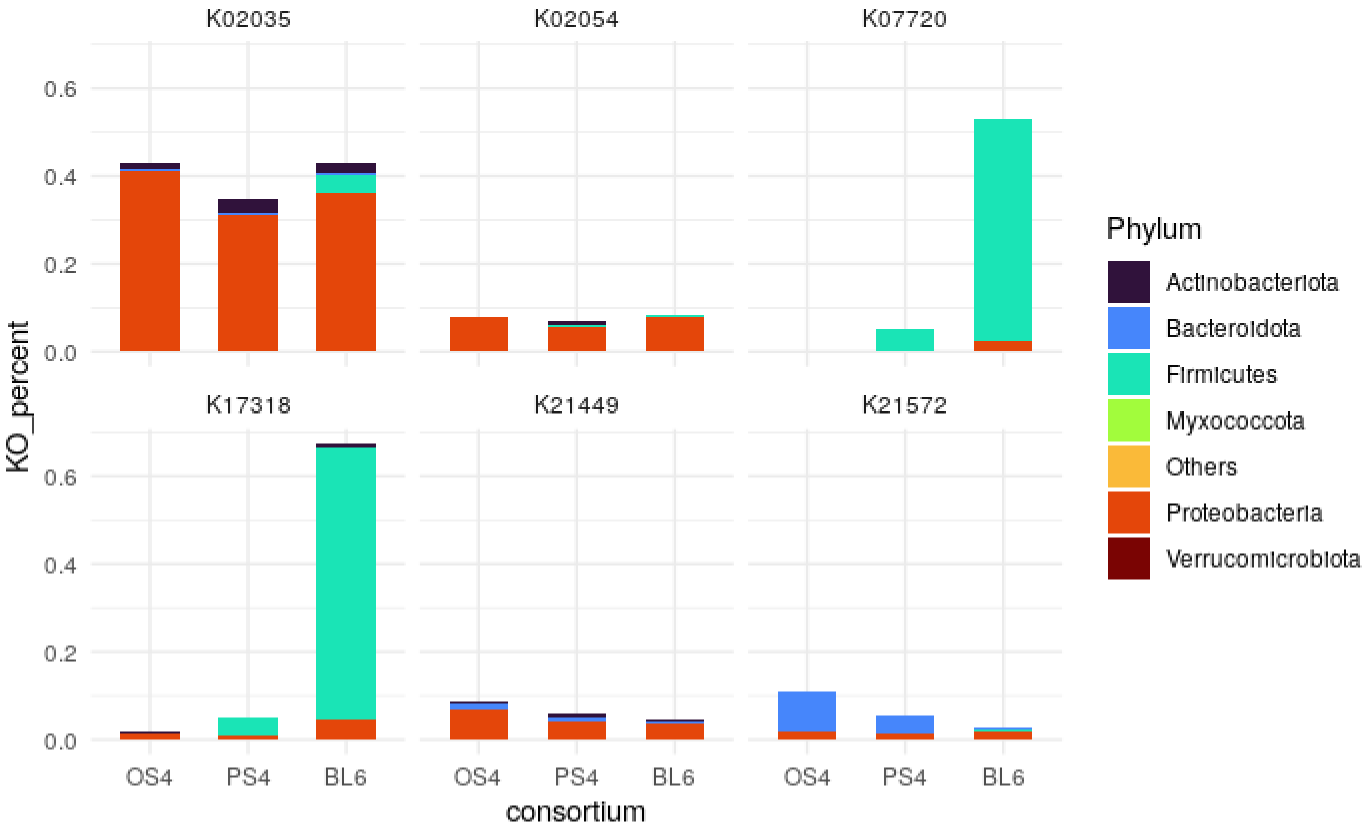

Figure S13. Dependence of inverted Simpson values and values of coefficient of variance for the KEGGs in different consortia

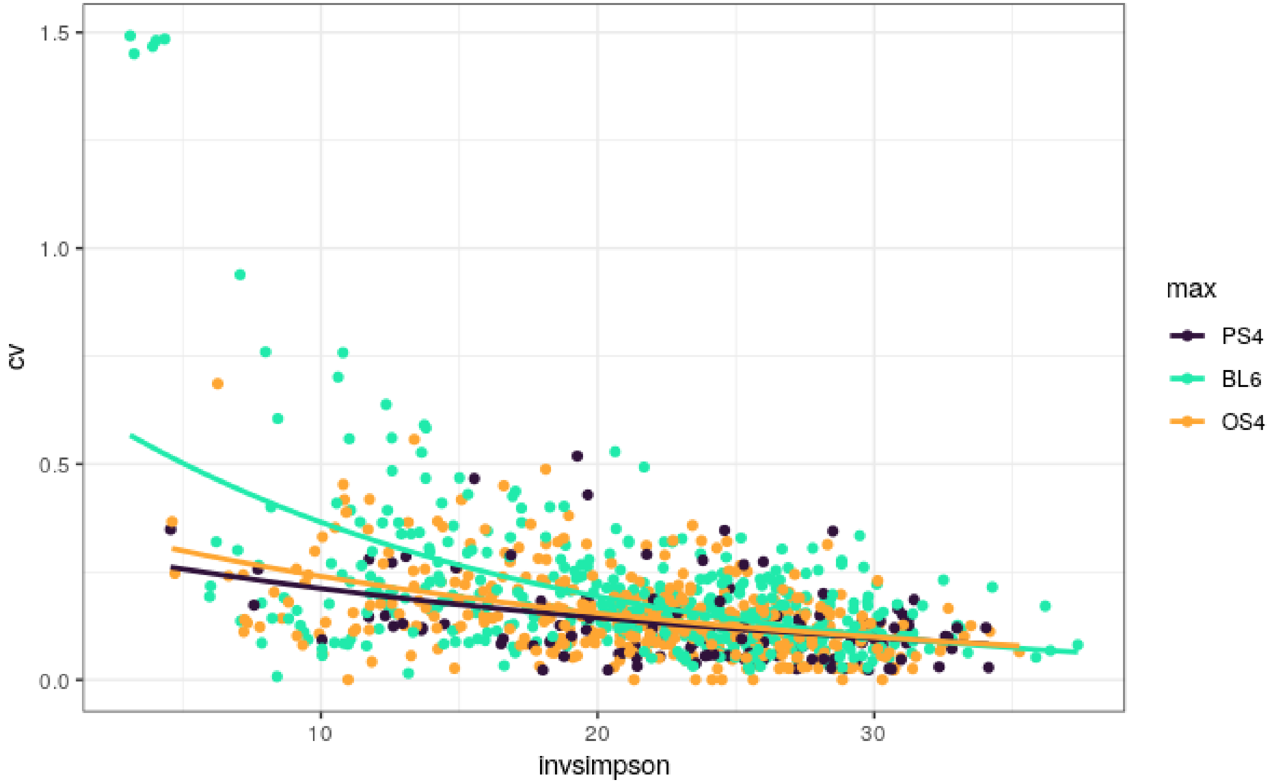

Supplement: Supplementary file 1 [file ijms-23-10779-s001.zip › gladkov_supplement_figures.pdf]
