# Supplementary material for: The Structure of Stable Cellulolytic Consortia Isolated from Natural Lignocellulosic Substrates
Source: Int J Mol Sci. 2022 Sep 15;23(18):10779. doi: 10.3390/ijms231810779 (PMC9501375; doi:10.3390/ijms231810779)
Supplement: Supplementary file 1 [file ijms-23-10779-s001.zip › gladkov_supplement_krona.html]

Javascript must be enabled to view this page.

magnitude
magnitudeUnassigned

14
26
29
46

1899158915191681

5455

0
1

1
0

0
1

1
0

1
0

0
1

1

1893158515141676
46102035

2
0

1
0

1
0

1
0

0
1

1

1
0

0
1

1
0

0
1

1

3194
001

001
3193

00
26

1
0

1
0

1

00
16

0
1

1

1
0

1

0
1

1

0
1

1

0
2

2

1
0

1

1
0

1
0

0
1

1

85
0

0
2

1
0

1

0
1

1

0
83

5
83

2

38

6

3

13

8

3

5

00
11

1
0

0
1

1

0
1

0
1

1

0
1

1
0

1
0

1
0

0
1

1

00
11

1
0

1
0

0
1

1
0

1

0
1

0
1

0
1

0
1

1

1184244
0000

1184244
0003

1
0

0
1

0
1

1

0
1

1
0

0
1

1

184239
000

0020
184239

1
0

1

40
0

40

1
0

1

3
0

3

4
1

1

1

1

1
0

1

0
4

3

1

5
0

1

1

1

1

1

1
0

1

12
34

1

1

1

3

1

1

1

1

1

1

1

1

1

2

1

1

1

2

00
8381

2

1

1

1

2

1

154

2

826

2

5

1

1

2

0
2

2

0
1

1

0
1

1

0
7

1

3

1

1

1

1
0

1

2
0

1

1

0
3

1

2

9
0

9

16
2

1

1

1

1

4

5

1

0
2

1

1

2
0

1

1

0
1

1
0

1
0

1

000
1532

0
1

1
0

0
1

1
0

1

00
153

153
00

143
20

1
0

1

0
1

1

00
83

83

1
0

1

1
0

1

0
1

0
1

1

0
1

1
0

0
1

1

1
0

0
1

1
0

1
0

0
1

1

2
0

2
0

1
0

0
1

0
1

1

0
1

0
1

1
0

1

246135131102
1000

0
1

0
1

0
1

0
1

1

0
1

0
1

1
0

1
0

1

8200
230135130102

711
100

2
0

0
1

1

0
1

1

00
11

00
11

1

1

1
0

0
1

1

21
00

0
1

1

1
0

1

0
1

1

95316
1000

114
000

1

1
0

1

13
00

3

1

75212
0000

0
1

1

11
00

1

1

1
0

1

00
11

1

1

0120
2511

1

4

1

1

1

1

1

1

1

1

1

1

1

1

1

1

1

1

1

1

1

2

1

1

1

3

1

1

1

1

1

1

1

1

1

1

1
0

1

0
1

1

0000
367410885

357410885
6210

00
22

1

11

1

1
0

1

0
1

1

11
00

1

1

0
1

1

0
1

1

1
0

1

1
0

1

2
0

1

1

2
0

1

1

716106
020

49

13105

111

11

185
00

184

1

2
0

2

10
51

1

1

1

2

0
1

1

0
1

1

0
3

3

247
13

4

1

1

1

3

24

1

9

1

0
1

1
0

1

2
0

2
0

0
1

1

0
1

1

3232
0000

3232
0010

0
1

1

1111
0000

11

1

1

1
0

1

11
00

1

1

1
0

1

1672178
0000

00
22

11
00

1

1

00
11

1

1

418
100

0
1

1

00
27

1

1

7

0
1

1

0
1

1

157115
000

000
157115

15315

41

0
4

0
1

1

1
0

1

0
1

1

1
0

1

13
0

0
13

1
0

1
0

1

0
12

12
0

12

1
0

0
1

1
0

1
0

1

29
00

0
2

0
1

0
1

0
1

1

1
0

0
1

0
1

1

0
9

9
0

9
0

0
9

2

7

0
1

0
1

1
0

0
1

0
1

1

1427
000

0
1

1
0

0
1

1

30
417

35
0

6
34

6
0

1

2

1

1

1

1
0

1

0
1

1

0
11

1

1

2

3

4

0
1

1

4
0

3

1

1

0
1

1

0
1

1

1
0

1

0
1

1
0

1

4
0

4
0

0
1

1

0
2

2

1
0

1

00
33

33
10

0
1

1

1
0

1

0
3

3

1
0

0
1

1
0

1
0

1

2
0

2
0

2
0

0
1

1
0

1

0
1

1
0

1

000
215

000
215

215
000

1
0

0
1

1

1
0

0
1

1

01
24

1
0

1

21
00

1

2

1
0

1

0
6

0
5

0
5

0
5

5
1

4

1
0

0
1

1
0

0
1

1

1
0

1
0

0
1

0
1

0
1

1

121814228951038
5273

10434
900868762735

0
1

0
1

0
1

1

1
0

1
0

1
0

1

9313481
0000

9313480
1010

091
410376

1

1

1

10

1

1

1

1

1

1

1

1

1

3

1

9

1

11

11

26

1

1

1

11

132

1

1

1

11

1

1

1

1

27

2

21

1

0
1

1

11
00

11

1
0

1

00
11

1

1

0
19

1

6

9

1

1

1

1

10
21

11

27
00

5

1

1

1

1

000
111

1

1

1

0
1

0
1

1

1
0

1
0

0
1

1

00
117

00
117

14
0

11

1

1

1

13
00

1

1

1

1

00
21

00
21

00
21

1

11

000
1259

000
1259

1258
000

1

1

158

0
1

1

768754615440
17343

21010753128
1000

1010
20210752123

112

11

11

1

1

1

1

11

2

1

1

1

1

11

1611115

8

431

1

1

1

11

11499

1

51473

1
0

1

65
31

1

1

1

1

1

1

1

1
0

1

0
21

0
3

1

2

0
18

18

0
1

1
0

1

2012
19118820

0000
1113

1

13

1

00
11

1

1

0
1

1

11
00

1

1

111055
5172

1

2

1

1

1

1

1

1

3

11

1

1

3

1

1

1

3

1

11

1

2

1

1

81

1

1

1

2

1

1

2

1

1

2

1

2

1

2

41

1

1

1

1

1

1

21

1

3

2

1

3

2

4

3

2

1

1
0

1

1
0

1

377
30

2

2

71

2

81

53

5

22

1

00
440

440

1
0

1

0
1

0
1

1

227
01

00
11

1

1

00
12

1

1

1

0
1

1

19
0

8

11

3
1

1

1

0
1

0
1

1

0
1

1
0

1

0002
184405188215

2781
000

2441

34

113293543
51101

1

2

55592

19115

311

61

11

8

1

2

110

1162120

1

15

1

4

63

1

11

11

13

111

2

11

116

68112105168
1937

14234

27336931

6151024

58569

72

2

815615

1

612516

1822

00
11

1

1

0
1

1
0

1

0
1

0
1

1

0
1

0
1

1

1
0

1
0

1

11911
31423717845

21
00

1

2

0
1

1

000
1141

1141

212
000

12

2

4
0

1

1

1

1

11
00

1

1

100
2150

1

1

1

47

1

1

1
0

1

4302
171164

1

1

2

2

1

11

1

1

1

1

1

1

1

1

11

1

1

1

1

1

1

1

1

1

1

0
1

1

000
111

1

1

1

1
0

1

1
0

1

2
0

1

1

4221
0001

1

1

1

1

2

1

1

0
1

1

11
00

11

0
2

1

1

00
51

51

5813723
183151

11

11

1

1

1

111

1

11

413

1

11

1

14

1

1

1

1

2

11

14

1

1

332

1

1

1

1

1

1

1

1

1

1

3

1

2

1

1

1

1

1

1

2

1

2

11

11

11

1

210

41

1

1

1

1

1

1
0

1

725026
000

12

211

222

34

21

62441

1

0
1

1

1100
12213711

1

141221

1

211

17

1026

010
221

1

1

1

1

1221
0000

1

1

1

1

2

1
0

1

00
21

1

1

1

1
0

1

00
21

21
00

10
11

1

1

0000
911024129

1
0

1
0

1

000
4717

113
4717

133

1

1

1

2

12

1

1

1

32

87954111
0000

07
5110

1

4

1

1

29

3

1

12

3

2

1

1

2

1

1

29

16

62
20

1

1

2

1

1

1
0

1

76934
020

1

1

1

1

31

133

4

7573

1

3

0000
14114

4001
14114

0
1

1

1
0

1

1
0

1

1
0

1

1
0

1

1

11
00

1

1

0
1

1

211
000

1

11

1

1
0

1

0
1

1

1
0

0
1

1
0

1

22
00

22
00

1
0

1

1
0

1

2
0

2

6413
313552126300

0
1

0
1

1
0

1

0000
3536710117

1510
212806115

0
1

1

1655110
0000

1

161593

110

3

1

2

2

1

1
0

1

1
0

1

000
1062

1

1

2

1

101

1

1

11

0
2

1

1

0131
81992

1

2

2

1

1

1

1

12

82

1

10

2

1

11

84

1

61

1

1

148742
0000

148742
0000

2

2

148342

0
1

1
0

1
0

1

0
1

1
0

0
1

1

111851
1000

0
1

0
1

1

010
421

0
1

1

311
100

1

1

1

1

2
0

0
1

1

0
1

1

1
0

0
1

1

00
11

0
1

1

1
0

1

010
3171

1
14

12

1

0
1

1

311
100

1

111

1010
256160109177

1
0

0
1

1

0
1

1

0
1

1
0

1

48
00

0
1

1

1
0

1

46
00

46

6281
254156106169

94135
20

9

168

3

1

1

19

35

93

13

163

1

00
11

1

1

0
1

1

42
20

1

1

1

1

31
10

1

1

1

0
1

1

00
21

1

1

1

1

1
0

1

0
1

1

0
1

1

1
0

1

001
33160

3

21

1158

0
1

1

1
0

1

36711
0000

36711

0
1

1

1
0

1

00
132

131

1

1

158
10

1

56

1

3
1

1

1

1
0

1

1
0

1

0
2

1

1

20
941

2

7

6

1

9

3

33

1

2

2

12

2

4

2

11

1

1

3

1
0

1

1
0

1

0
1

1

0
1

1

0
1

1

1
0

1

0
1

1

1
0

0
1

0
1

1

1
0

1
0

0
1

1

000
311

1
0

0
1

1

2
0

1
0

1

0
1

1

11
00

1
0

1

0
1

1

1
0

0
1

0
1

1

0000
26259386

1
0

0
1

1
0

1
0

1

26259286
4000

0
3

1
0

1
0

1

1
0

0
1

1

1
0

1
0

1

331
30

1
0

1
0

1

1
0

1
0

1

0
2

2
1

1

10
261

50
221

9

5

31

2
0

1

1

1
0

1

0
1

1
0

0
1

1

0
1

1
0

0
1

1

21849186
0000

21849185
18089

6020
311334

24

211

54

14

412

48

21

452

121

000
4037

32

3735

0
33

33

000
512

512

4
1

2

1

646
011

1

23

312

11

20
52

12

1

1

000
321

321

000
212

1

1

1

1

1

42
00

41

1

000
321

1

11

21

4
0

4

0
2

1

1

1192
100

321

111

22

2

42

0
4

2

2

12545
105

16

212

116

8

6212

26

22
00

21

1

31113
300

2

121

33

1

711

42

311

6

1

2

0
1

1

422
100

12

11

2

1
0

1

0
1

0
1

1

00
21

2
0

0
1

1

1
0

1

1
0

0
1

1

0000
85523663

85523663
121134

0052
81568

01
37

1

02
23

1

1

1

1

1
0

1

1
0

1

41385
0061

0
1

1

02
15

1

1

1

1

1121
0001

1

1

1

1

2
1

1

2
1

1

00
11

1

1

070
1193

1

1

1

1

1

1

1

1

1

1

1

1

1

1

1

1

0
1

1
0

1

000
141

131
000

1

1

1

1

1

0
1

1

0
1

0
1

1

165
010

00
31

00
31

1

1

1

1

000
124

0
2

1

1

00
14

14

0
1

0
1

1
0

1

6
0

0
1

1
0

1

2
0

2
0

2

1
3

0
2

1

1

46213244
0071

00
11

00
11

1

1

11
00

00
11

11

6110725
0010

0
1

1

0
1

1

0
46

46

1
0

1

1
0

1

1
0

1

1
0

1

0
1

1

0
1

1

4524
00

95

104

1

1

21

45

189

519
202

1

1

1

1

1

1

1

1

2

1

351218
0000

1

311218
0000

9

3

1

3

2011

1

1

3

1

1

2

2

1

11

3
0

1

2

11
00

1
0

1

1
0

1

00
14

0
1

1

1
0

1

1
0

1

1
0

1

0
1

1

19
01

0
1

1

0
2

1

1

1
0

1

1
2

1

0
1

1

0
1

1

0
1

1

171181
0000

0000
171181

151171
6050

1

1

1

1

1

1

1

1

1

11

1

1

1

1

1

1

1

1

1

1

1

1

1
0

1

1

1
0

1

5
0

1
5

0
1

1

2
0

1

1

0
1

1

0
1

1
0

0
1

1
